# Integrative Analysis of Shared Pathogenic Genes and Potential Mechanisms in *Gardnerella vaginalis* and Persistent HPV16 Infection

**DOI:** 10.1155/mi/2582989

**Published:** 2025-06-05

**Authors:** Ye Li, Yue Wang, Xianhua Liu, Huifeng Xue, Liying Wang, Maotong Zhang, Pengming Sun

**Affiliations:** ^1^Laboratory of Gynecologic Oncology, Fujian Maternity and Child Health Hospital, College of Clinical Medicine for Obstetrics and Gynecology and Pediatrics, Fujian Medical University, Fuzhou 350001, Fujian, China; ^2^Fujian Key Laboratory of Women and Children's Critical Diseases Research, Fujian Maternity and Child Health Hospital (Fujian Women and Children's Hospital), Fuzhou 350001, Fujian, China; ^3^Fujian Clinical Research Center for Gynecological Oncology, Fujian Maternity and Child Health Hospital (Fujian Obstetrics and Gynecology Hospital), Fuzhou 350001, Fujian, China; ^4^Department of Pathology, Fujian Maternity and Child Health Hospital, Fuzhou 350001, Fujian, China; ^5^Medical Center of Cervical Disease and Colposcopy, Fujian Maternity and Child Health Hospital, Fuzhou 350001, Fujian, China

**Keywords:** *Gardnerella vaginalis*, IFIT1, molecular mechanisms, persistent HPV16 infection, RSAD2

## Abstract

Bacterial vaginosis, often accompanied by *Gardnerella vaginalis* (GV) overgrowth, is associated with persistent high-risk human papillomavirus (HR-HPV) infection, particularly HPV16. This study integrated transcriptomic data from in vitro GV infection experiments and a GEO dataset (GSE75132) of HPV16 persistence to elucidate shared pathogenic mechanisms. Differential expression analysis identified 4115 genes modulated by GV infection and 861 by HPV16 persistence, with 74 common differentially expressed genes (DEGs) displaying consistent trends. Enrichment analyses revealed that these DEGs participate in metabolic pathways, immune defense, host–pathogen interactions, and carcinogenesis. Protein–protein interaction networks and Random Forest (RF) feature selection pinpointed radical S-adenosyl methionine domain containing 2 (RSAD2) and Interferon-induced protein with tetratricopeptide repeats 1 (IFIT1) as central hub genes. Upstream transcription analysis identified the homer_AGTTTCAGTTTC_ISRE motif and established a ceRNA network involving hsa-miR-654-5p, IFIT1/RSAD2, and lncRNAs. Mendelian randomization (MR) and colocalization analyses linked RSAD2 downregulation to an increased risk of cervical carcinoma in situ (rs2595163, PPH4 = 0.62), while ROC analysis demonstrated strong diagnostic potential for the combined hub gene expression. Notably, single-cell transcriptomics revealed distinct RSAD2 and IFIT1 expression patterns in immune and epithelial cells during the progression from HPV infection to cervical cancer. Collectively, these findings support RSAD2 and IFIT1 as promising biomarkers and therapeutic targets for HPV-related cervical lesions.

## 1. Introduction

Cervical cancer is the fourth leading cause of cancer-related deaths in women worldwide, with ~660,000 new cases and 350,000 deaths reported in 2022 [1]. Approximately 70% of these cases result from persistent infections with high-risk human papillomaviruses (HR-HPV) genotypes 16 and 18 [2]. Globally, among women who have not been vaccinated, HPV16 is the most prevalent genotype associated with cervical cancer, cervical intraepithelial neoplasia (CIN), and both normal and abnormal cytology [3]. Women with CIN II have a 47% chance of progressing to CIN III or higher within 2 years if HPV16 infection remains unresolved [4].

Despite the high prevalence of HPV infection [5], its relatively low pathogenicity highlights the complexity of its disease mechanisms. Both HR-HPV clearance and disease regression have been linked to the composition of the cervicovaginal microenvironment, influenced by the host immune system and vaginal microbiota [6]. A shift from a *Lactobacillus*-dominant vaginal flora to a more diverse microbial environment, particularly with an overgrowth of anaerobic bacteria such as *Gardnerella*, *Atopobium*, *and Prevotella* (hallmarks of bacterial vaginosis), is closely associated with increased susceptibility to HR-HPV infection and persistence [7–9]. Conversely, persistent HR-HPV infection may further disrupt the composition of the cervicovaginal microbiome [10].

Increasing clinical evidence suggests that excessive proliferation of *Gardnerella vaginalis* (GV) within the bacterial vaginosis-associated microbiota is closely linked to persistent HPV infections [9, 11–14]. For example, Yang et al. [13], demonstrated a decrease in the proportion of *Lactobacillus* and an increase in *Gardnerella* among women infected with HPV16. Furthermore, research by Belletti et al. [11] revealed that increased vaginal microbial diversity is detrimental to the clearance of HPV16, particularly in individuals with a high GV burden, who exhibit reduced HPV16 clearance rates. Several factors may contribute to this phenomenon, including GV overgrowth, which disrupts the *Lactobacillus* dominance in the vagina, thereby creating a favorable environment for HPV persistence [6, 15–17]. Additionally, biofilms formed by GV can protect HPV from immune surveillance [18, 19]. Moreover, inflammation and metabolic byproducts induced by GV compromise the cervical protective barrier, reducing the immune system's efficiency in clearing HPV, thereby facilitating HPV infection and its persistence [13, 20, 21].

Although numerous studies have demonstrated that GV overgrowth is strongly linked to persistent HPV16 infection, the molecular mechanisms underlying their co-infection remain poorly understood. This study aims to uncover their shared pathological mechanisms by identifying common transcriptomic features of GV infection and persistent HPV16 infection.

During the experimental process, we first obtained transcriptome data associated with GV and integrated it with microarray data from individuals with persistent HPV16 infection. By identifying differentially expressed genes (DEGs), merging the two datasets, and applying random forest (RF) and protein–protein interaction (PPI) network analysis, we identified key hub genes that are commonly involved in both GV infection and persistent HPV16 infection. To further investigate these hub genes within the co-infection setting, we conducted upstream regulatory analysis to explore their transcriptional regulation and constructed a competing endogenous RNA (ceRNA) network. Additionally, we performed single-gene gene set enrichment analysis (GSEA) and correlation analysis to elucidate the biological functions potentially influenced by these genes. The diagnostic potential of the hub genes was evaluated using ROC analysis, while their clinical significance in cervical carcinogenesis and prognosis was assessed through Mendelian randomization (MR) analysis and survival analysis. Finally, single-cell analysis was conducted to examine expression pattern alterations during cervical carcinogenesis, providing insights into the clinical implications of their dysregulation.

Overall, this study, by identifying hub genes associated with both GV and HPV16 persistent infection, comprehensively explores the molecular underpinnings of this co-infection. This is the first systematic investigation of GV and HPV16 co-infection from a comorbidity perspective, paving a new path for research into the mechanisms of transformation from cervicitis to cancer and laying a foundation for future research and clinical treatment.

## 2. Materials and Methods

### 2.1. Cell Lines

Siha (ATCC HTB-35) and HaCaT (SNL-163, Sunncell, Wuhan, China) cells were cultured in Dulbecco's Modified Eagle Medium, while Caski (ATCC CRL-1550) cells were cultured in RPMI-1640; these media were supplemented with 10% fetal bovine serum and 1% penicillin–streptomycin. All cultures were maintained at 37°C in a humidified 5% CO_2_ atmosphere. Cells were passaged every 2–3 days at ~80% confluence using 0.25% trypsin-EDTA.

### 2.2. Bacterial Strains and Culture Conditions

*G. vaginalis* (ATCC 14018) was rehydrated using New York City (NYC) III broth according to the manufacturer's instructions. A volume of 20 μL of the bacterial suspension was streaked using a three-zone streaking method onto sterilized Columbia Blood Agar (Macklin) and placed in an anaerobic bag with a chemical absorbent. The setup was then incubated at 37°C for 48 h. Post-incubation, transparent colonies measuring 1–2 mm in diameter were observed and subsequently subcultured onto fresh Columbia Blood Agar under similar conditions.

### 2.3. Establishment of a Co-culture System Between GV and Host Cells

**Figure 1:**
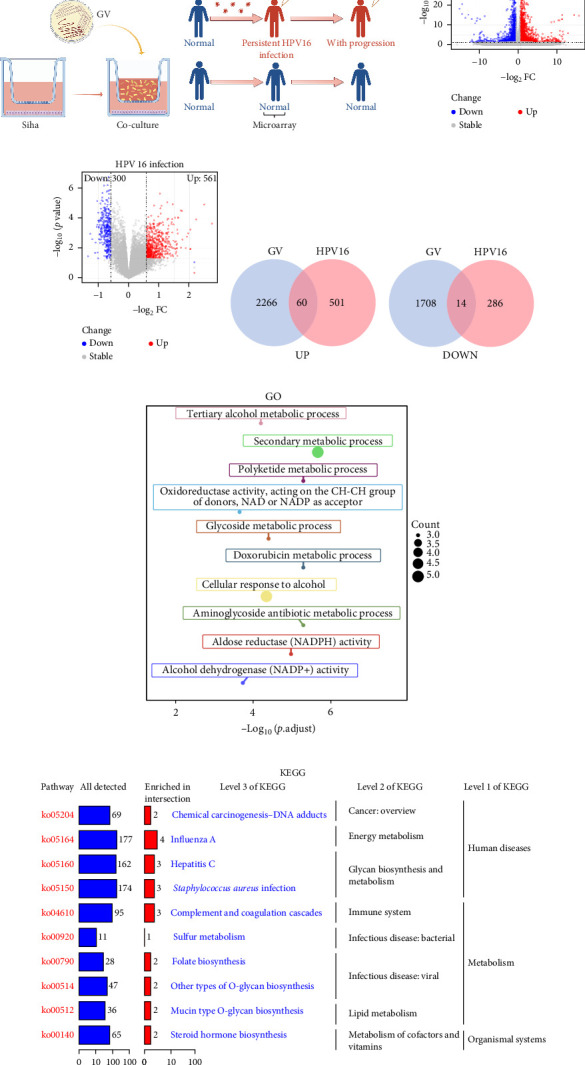
DEGs co-regulated by GV infection and persistent HPV16 infection, and their enrichment analysis. (a) GV co-culture model diagram; (b) Grouping information for persistent HPV16 infected populations; (c) Volcano plot of DEGs regulated by GV infection; (d) Volcano plot of DEGs regulated by persistent HPV16 infection; (e) Venn diagram of common DEGs (co-regulated by GV infection and persistent HPV16 infection), showing the commonly upregulated genes; (f) Venn diagram of common DEGs, showing the commonly downregulated genes; (g) Bubble chart of GO enrichment analysis for common DEGs. The top 10 most significant terms (sorted by *p*-value) are shown; (h) Treemap of KEGG enrichment results for the common DEGs. DEGs, differentially expressed genes; GV, *Gardnerella vaginalis*; KEGG, Kyoto Encyclopedia of Genes and Genomes.

Cells were seeded at ~2.5 × 10^5^ cells per well in a 6-well plate with complete medium. Post-adhesion, the wells were rinsed with sterile saline and fitted with invasion chambers having a pore size of 0.1 mm. By utilizing the invasion chamber to physically isolate GV from direct contact with host cells, acute cell death due to direct bacterial toxicity was avoided, enabling indirect interaction between the two through soluble factors in the culture medium (such as metabolites, extracellular vesicles, etc.). GV was prepared by swabbing colonies from Columbia Blood Agar, resuspending them in sterile saline to a final concentration of 6 × 10^8^ CFU/mL as measured by OD550nm turbidimetry, and centrifuging at 13,000g for 5 min. The bacterial pellet was then resuspended in penicillin-streptomycin-free medium enriched with 10% fetal bovine serum. For co-culturing, ~1 × 10^8^ CFUs/mL of the bacterial suspension were added along the chamber walls and incubated at 37°C in 5% CO_2_ for 24 h. (Figure 1a). After incubation, the supernatant containing GV was collected and spread on Columbia Blood Agar plates, which were then incubated under anaerobic conditions for 24–48 h. The appearance of GV colonies confirmed the continuous interaction between viable GV and the cells during the co-culture process.

### 2.4. Transcriptome Sequencing of GV Infection

In this study, Siha cells infected with GV were used as the experimental group, while Siha cells without GV infection served as the control group. Six biological replicates were included for each group, and transcriptome sequencing analysis was performed. Total RNA was extracted from Siha cells using Trizol, quality-checked via Bioanalyzer 2100 with RNA 6000 Nano LabChip Kit (Agilent), and only samples with RIN >7.0 were selected. mRNA from 5 µg of total RNA was isolated using Dynabeads Oligo (dT) (Thermo Fisher), fragmented at 94°C for 5–7 min with the magnesium RNA Fragmentation Module (NEB), and reverse transcribed using SuperScript II Reverse Transcriptase (Invitrogen). The cDNA underwent second-strand synthesis with *E. coli* DNA Polymerase I, RNase H, and dUTP Solution (NEB/Thermo Fisher). Libraries, prepared using AMPure XP beads for size selection and adaptor ligation, were PCR amplified and assessed to ensure an average insert size of 300 ± 50 bp, and sequenced on the Illumina NovaSeq 6000 with 2 × 150 bp paired-end reads. Transcriptome data, derived from RNA-Seq results of the same batch, were standardized using the FPKM method, processed with the StringTie pipeline, and subsequently analyzed for differential expression using R's DESeq2 package, thereby requiring no additional batch correction.

### 2.5. Differential Expression and Functional Enrichment Analysis

The primary dataset, GSE75132 (GPL570 platform), was derived from the Danish HPV16 persistent infection cohort and included 20 samples from individuals exhibiting cervical pathological progression during follow-up, with 11 healthy controls [22]. In the HPV16 persistent infection expression profile, DEGs were identified using the limma package after normalizing the microarray data with the LOWESS method to correct technical biases, median-normalizing to 10,000, and converting values to ratios relative to HPV-negative samples as the baseline. The selection criteria were set as *p* < 0.05 and |log_2_ FC| ≥ 0.585. Volcano plots were generated with ggplot2 to visualize the DEGs, and Venn diagrams created with ggvenn to illustrate the number of genes with consistent expression trends between the HPV16 persistent infection and GV infection samples. Subsequently, gene ontology (GO) and Kyoto Encyclopedia of Genes and Genomes (KEGG) enrichment analyses of the consistently expressed DEGs were performed with clusterProfiler, and the results were visualized using ggplot2, considering *p* < 0.05 as significant.

### 2.6. Feature Gene Selection by Using RF

The randomForest R package was employed to identify feature genes associated with GV and persistent HPV16 infections from the common DEGs. The model was configured with 500 trees, and the *mtry* parameter was set to the square root of the number of genes. Genes were ranked in descending order based on MeanDecreaseAccuracy values. Genes with an importance value above 1.2 were deemed significant feature genes. Subsequently, the intersection of feature genes associated with GV infection and persistent HPV16 infection was used to identify common feature genes.

### 2.7. Mapping PPI Networks and Identifying Hub Genes

Interactions among common DEGs were explored using the STRING database (https://cn.string-db.org/), with a median confidence threshold of >0.4 for constructing interaction networks. The resulting PPI networks were visualized in Cytoscape (v3.10.1). To identify hub genes, nine algorithms (Betweenness, Stress, Closeness, Degree, DMNC, EPC, MCC, MNC, and Radiality) from the CytoHubba plugin in Cytoscape were applied to score the common DEGs. The top 10 genes from each algorithm were compared, and the overlapping key genes were selected, and the intersection of these key genes with the common feature genes used to determine the hub genes.

### 2.8. Transcription Enrichment Analysis

Enrichment analysis of transcription factors (TFs) and binding motifs linked to hub genes was performed using the RcisTarget R package. Significant motifs with a Normalized Enrichment Score (NES) greater than 3 were identified. TF data related to these motifs were extracted, and their expression levels were analyzed using the area under the curve (AUC) method.

### 2.9. Construction of the ceRNA Network

To construct the ceRNA network of hub genes, regulatory miRNAs for the hub genes were first predicted using the miRwalk database (http://mirwalk.umm.uni-heidelberg.de/). Relevant miRNAs associated with HPV infections were then identified in the HMDD database (http://www.cuilab.cn/hmdd). Interactions between these miRNAs and lncRNAs were predicted using the ENCORI database (https://rnasysu.com/encori/index.php). Finally, the regulatory network comprising lncRNAs, miRNAs, and hub genes was visualized in Cytoscape (v3.10.1).

### 2.10. GSEA and Correlation Analysis With Phenotypic Genes

To identify significantly enriched pathways associated with hub genes, Spearman's correlation coefficients were calculated between the hub genes and all other genes in the expression matrix using the corollary.test function in the R stats package. Genes were ranked based on these coefficients, and the resulting list was analyzed for GSEA using the clusterProfiler package, targeting GO and KEGG pathways. Pathways with an adjusted *p*-value < 0.05, false discovery rate (FDR) <0.25, and absolute normalized enrichment score (|NES|) ≥ 1 were deemed significantly enriched. Subsequently, to explore relationships between the hub genes and potential phenotypic genes, the top 100 phenotype-related genes were retrieved from the GeneCards database (https://www.genecards.org/) based on relevance scores linked to phenotypes from the significantly enriched pathways. These phenotypic genes were then subjected to Spearman's correlation analysis with the hub genes using the corollary test function, and genes with an FDR-adjusted *p*-value < 0.05 were considered significantly correlated.

### 2.11. Expression Analysis and Diagnostic Evaluation of Hub Genes

The expression differences of hub genes (radical S-adenosyl methionine domain containing 2 [RSAD2] and interferon-induced protein with tetratricopeptide repeats 1 [IFIT1]) between control and disease groups were validated using the GSE137964, GSE234836, and GSE129159 datasets. Differential expression analysis was performed using “Limma” package for microarray data and “DESeq2” for RNA-seq data. The diagnostic performance of these hub genes was evaluated by constructing receiver operating characteristic (ROC) curves with the “ROCR” package, with an aAUC > 0.7 considered satisfactory. Furthermore, a multi-gene diagnostic model incorporating RSAD2 and IFIT1 was developed using logistic regression.

### 2.12. MR and Colocalization Analysis

Cis-eQTLs for hub genes were sourced from the eQTLGen consortium (https://eqtlgen.org/) and genome-wide association study (GWAS) four stages of cervical lesion (low-grade squamous intraepithelial lesion [LSIL], high-grade squamous intraepithelial lesion [HSIL], cervical carcinoma in situ [CIS], and squamous cell carcinoma [SCC]) were obtained from the FinnGen consortium [23] (https://r10.finngen.fi/) are detailed in Supporting Information 1: Table S5. MR analyses between hub genes and four stages of cervical lesions were conducted using the TwoSampleMR package in R. The analysis employed inverse variance weighting (IVW) to estimate causal effects and MR-PRESSO to detect and adjust for horizontal pleiotropy. Heterogeneity was assessed using the Cochran *Q* statistic in both the IVW and MR-Egger methods, with the MR-Egger intercept also utilized to test for pleiotropy. Summary-data-based MR (SMR software: http://cnsgenomics.com/software/smr/) was conducted to determine if single nucleotide polymorphism (SNP) effects on cervical lesions were mediated through Hub gene expression, using the 1000 Genomes European reference to calculate linkage disequilibrium. Colocalization analysis identified shared causal variants between hub gene expression and cervical lesions. Significance thresholds were set at *p*_adj_ (IVW method, FDR adjustment) <0.05, *p*_SMR_ < 0.05, *p*_HEIDI_ > 0.05, with shared causal variants determined by a PPH4 > 0.05 using the Coloc R package.

### 2.13. Survival Analysis of Hub Genes

Gene expression data and clinical survival information for cervical cancer (TCGA-CESC) were retrieved from the TCGA database (https://portal.gdc.cancer.gov/), including three normal samples and 296 tumor samples. The samples were stratified into groups based on the expression levels of the hub gene using the “survival” package in R. Kaplan–Meier survival curves were generated using the “survminer” package, with statistical significance set at *p* < 0.05.

### 2.14. Single-Cell RNA Sequencing Analysis of Hub Gene Expression

This study downloaded the GSE168652 dataset (1 HPV16-positive cervical cancer sample and 1 normal cervical sample) and the GSE171894 dataset (2 HPV16-positive and 2 HPV-negative squamous cell carcinoma samples) from the GEO database and analyzed them using R (version 4.4.0). Data preprocessing was performed with the Seurat package, including quality control, normalization, and identification of highly variable genes. The Harmony package was used to integrate samples and correct batch effects. Subsequently, the FindClusters function was applied to cluster cells from GSE168652 into 12 clusters and those from GSE171894 into 14 clusters. Cell subpopulations were annotated using marker genes from the easybio package and the CellMarker2.0 database. Finally, the expression patterns of hub genes across cell clusters were visualized with DotPlot and FeaturePlot, and their differential expression between sample groups was assessed using VlnPlot.

### 2.15. Real-Time PCR

RNA was extracted using TRIzol Reagent (TIANGEN), and cDNA was synthesized with GoScript Reverse Transcription Mix, Oligo (dT) (PROMEGA). Real-time PCR (RT-PCR) was then performed to quantify the expression levels of IFIT1 and RSAD2 using Eastep QPCR Master Mix (2×) (PROMEGA). Primer sequences for these genes are provided in Supporting Information 1: Table S13.

### 2.16. Statistics

Statistical differences between groups were evaluated using Student's *t*-test. One-way ANOVA was used for comparisons among multiple groups, followed by Tukey's test for pairwise comparisons. *p<*0.05 was considered statistically significant. Statistical analyses were conducted using R version 4.3.1 and GraphPad Prism version 9.5.

## 3. Results

### 3.1. DEGs Co-regulated by GV Infection and Persistent HPV16 Infection and Their Enrichment Analysis

**Figure 2:**
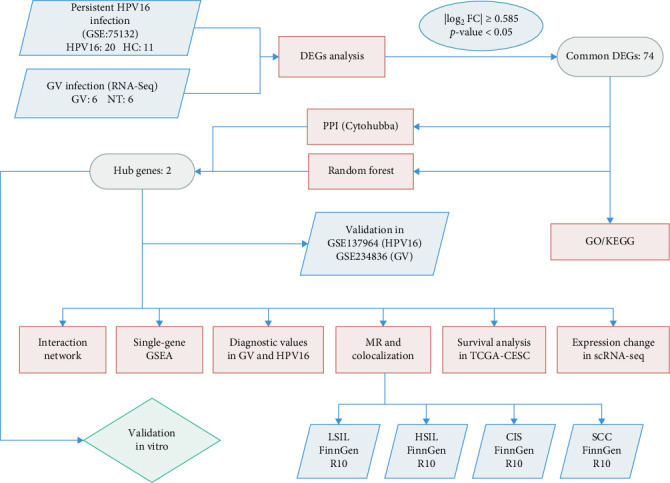
Research process flowchart. CIS, Carcinoma in situ; DEGs, differentially expressed genes; GO, gene ontology; GSEA, gene set enrichment analysis; GV, GV, *Gardnerella vaginalis*; HC, healthy control; HSIL, high grade squamous intraepithelial lesion; KEGG, Kyoto Encyclopedia of Genes and Genomes; LISL, low grade squamous intraepithelial lesion; MR, Mendelian randomization; SCC, Squamous cell carcinoma; scRNA-seq, single-cell RNA sequencing data; TCGA-CESC, data sourced from The Cancer Genome Atlas-Cervical squamous cell carcinoma and endocervical adenocarcinoma.

The specific process of this study is shown in Figure 2. DEGs influenced by GV infection and persistent HPV16 infection were identified using a threshold of |log2FC| ≥ 0.585 and *p* < 0.05. GV infection altered the expression of 4115 genes (1742 downregulated, 2373 upregulated; Figure 1c), while persistent HPV16 infection affected 861 genes (300 downregulated, 561 upregulated; Figure 1d). Integration of these datasets revealed 74 DEGs with concordant expression patterns (Figure 1e,f), as a key gene set potentially implicated in co-infection pathogenesis. Subsequently, enrichment analysis revealed that these 74 DEGs were significantly enriched in GO terms related to metabolic pathways, NADP + enzyme activity, and cellular responses. Notably, significant enrichment was also observed in secondary metabolism, a process often associated with microbial resistance and adaptation (Figure 1g, *p* < 0.05). Furthermore, KEGG pathway enrichment analysis indicated that these common DEGs were also significantly enriched in pathways related to folate and steroid hormone biosynthesis, pathogen infection, immune defense, disease resistance, and chemical carcinogenesis (Figure 1h, *p* < 0.05).

### 3.2. Uncovering Hub Genes Associated With GV and Persistent HPV16 Infection

**Figure 3:**
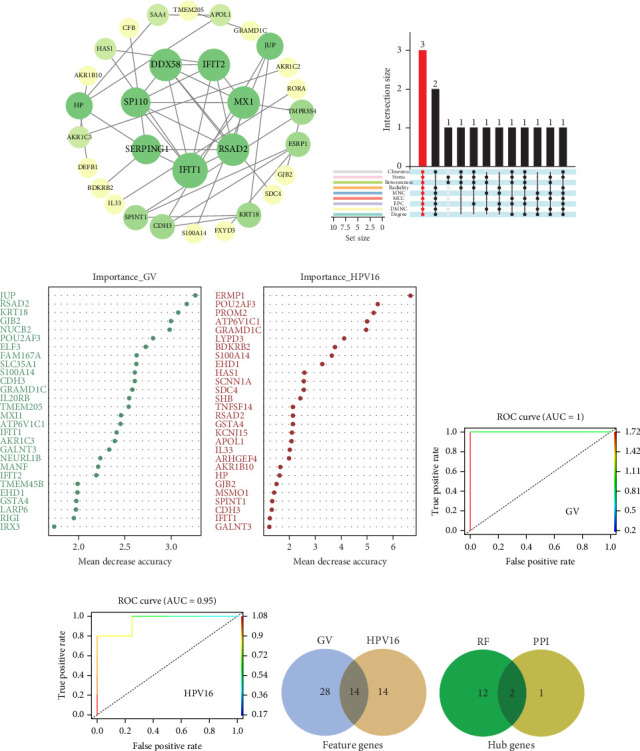
Identification of hub genes co-regulated by GV and persistent HPV16 infection (a) PPI network of the common DEGs; (b) Upset Venn diagram of the top 10 genes ranked by nine Cytohubba algorithms. The red bars represent genes consistently ranked within the top 10 across all nine algorithms (key genes); (c) Feature genes associated with GV infection; (d) Feature genes associated with persistent HPV16 infection; (e) AUC curve of the Random Forest model for predicting GV infection; (f) AUC curve of the Random Forest model for predicting persistent HPV16 infection; (g) Venn diagram of common feature genes shared by GV and persistent HPV16 infection; (h) Venn diagram showing the intersection between the common feature genes identified by random forest for GV and persistent HPV16 infections and the key genes ranked in the top 10 by all nine Cytohubba algorithms in the PPI network. AUC, area under the curve; DEGs, differentially expressed genes; GV, *Gardnerella vaginalis*; PPI, protein–protein interaction.

On the one hand, we utilized nine algorithms within the Cytohubba plugin (Betweenness, Stress, Closeness, Degree, DMNC, EPC, MCC, MNC, and Radiality) to score and rank the 74 common DEGs (Figure 3a and Supporting Information 1: Table S1). The top 10 DEGs from each algorithm were selected, and the intersection of these nine sets yielded 3 DEGs identified as crucial genes occupying central positions within the PPI network (Figure 3b). These three DEGs are referred to as core DEGs.

On the other hand, a Random Forest model was employed for feature selection, extracting feature genes associated with GV infection and persistent HPV16 infection from the gene expression matrix, respectively. With an importance threshold set greater than 1.2, 42 GV infection-related feature genes (Figure 3c) and 28 HPV16 infection-related feature genes (Figure 3d) were identified. ROC curves demonstrated that the GV infection model achieved an AUC of 1 (Figure 3e), and the persistent HPV16 infection model reached 0.95 (Figure 3f). The intersection of these two feature gene sets yielded 14 common feature genes associated with both GV and persistent HPV16 infection (Figure 3g).

Finally, the intersection of these 14 common feature genes with the previously identified 3 core DEGs resulted in 2 hub genes: IFIT1 and RSAD2 (Figure 3h). These two genes not only reflect the characteristics of GV infection and persistent HPV16 infection but also reside at the core of gene expression regulation. Therefore, IFIT1 and RSAD2 are considered key regulatory factors during co-infection.

### 3.3. Upstream Transcription and ceRNA Network Analysis of Hub Genes

**Figure 4:**
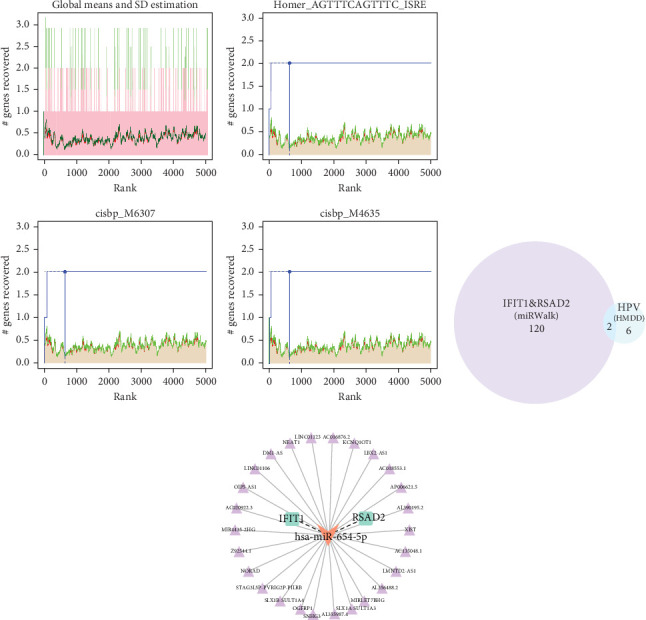
Upstream transcriptional regulation analysis of hub genes. (a) Displays the top three motifs by highest AUC values. Red line shows each motif's average recovery curve, green line is the average plus standard deviation, and blue line represents the recovery curve for the current motif. The maximum distance between the current motif and green line marks the peak enrichment level; (b) Venn diagram of miRNAs targeting hub genes from the miRwalk database and HPV-related miRNAs from the HMDD database; (c) ceRNA network of hub genes, with orange representing miRNAs, green representing mRNAs, and purple representing lncRNAs. AUC, area under the curve; HPV, human papillomaviruses.

Upstream transcription analysis of the hub genes IFIT1 and RSAD2 showed that the motif with the highest normalized enrichment score (NES) is HOMER_AGTTTCAGTTTC_ISRE (Figure 4a), which is significantly enriched in both genes. This observation is consistent with the established roles of IFIT1 and RSAD2 in antiviral responses, those driven by interferon stimulation. Further investigation revealed that the enriched TFs predominantly belong to the IRF family, with IRF4 and IRF8 being the most prominent. Additionally, TEAD4 and ZBTB18 were frequently identified in motif-TF TF annotations, suggesting their potential regulatory roles in the regulatory network (refer to Supporting Information 1: Table S2).

Building on these insights, this study further explored the upstream ceRNA network of IFIT1 and RSAD2. Using the miRwalk database, 122 miRNAs were predicted to target both genes, enabling the construction of a miRNA–mRNA interaction network (Supporting Information 2: Figure S1). Subsequent analysis using the HMDD database, which specializes in disease-related miRNAs, identified eight HPV-related miRNAs. Among these, hsa-miR-34a-3p and hsa-miR-654-5p were found to regulate the mRNA expression of IFIT1 and RSAD2 (Figure 4b). Based on predictions from the ENCORI database, a ceRNA network was constructed incorporating hsa-miR-654-5p and 26 lncRNAs, referred to as the lncRNA_hsa-miR-654-5p_IFIT1/RSAD2 network (Figure 4c).

### 3.4. Exploring the Potential Roles of Hub Genes in Co-infection

**Figure 5:**
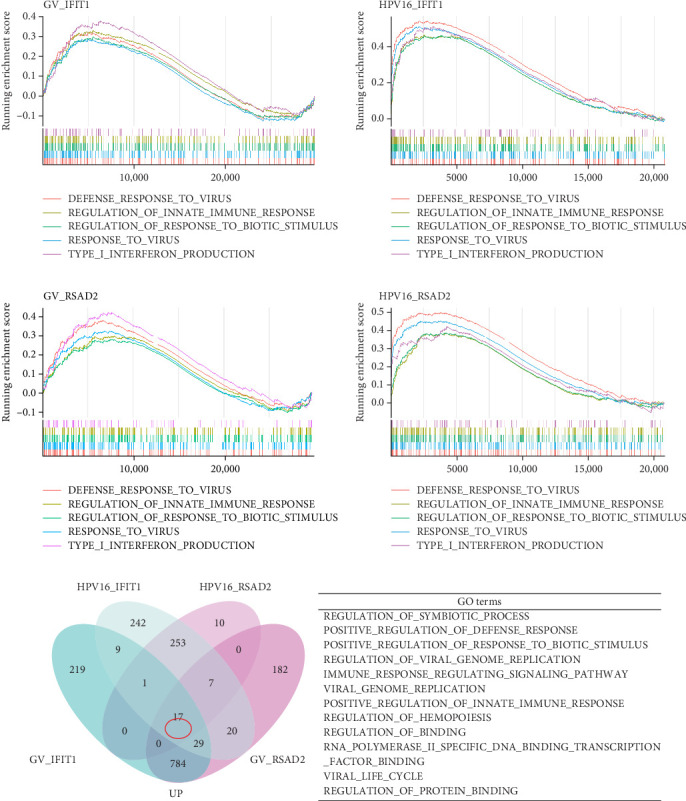
Single-gene GSEA enrichment analysis of hub genes (GO gene sets). GSEA enrichment analysis of IFIT1 in the GV infection dataset (a) and the persistent HPV16 infection dataset (b), with five GO terms co-enriched by IFIT1 and RSAD2 across both datasets highlighted in the plots; GSEA enrichment analysis of RSAD2 in the GV infection dataset (c) and the persistent HPV16 infection dataset (d), with the same five GO terms highlighted; (e) Venn diagram of GO terms commonly enriched by both IFIT1 and RSAD2 across the two datasets; (f) Detailed list of the remaining 12 GO terms consistently enriched by both genes in both datasets. All GO terms shown in the plots are upregulated entries. GO, gene ontology; GV, *Gardnerella vaginalis*; GSEA, gene set enrichment analysis.

To investigate the biological functions and signaling pathways co-regulated by the hub genes IFIT1 and RSAD2 during GV and HPV16 co-infection, we performed GSEA for each gene across both datasets. The enrichment results from the two datasets were combined, with a focus on terms enriched by both IFIT1 and RSAD2. Integrated GO analysis identified 17 significantly enriched terms, predominantly associated with viral defense, immune response, response regulation, cellular and molecular function modulation, and host-pathogen interactions.  Figure 5a–d highlights five GO terms that were simultaneously upregulated by RSAD2 and IFIT1 in both the GV infection and HPV16 infection datasets. A Venn diagram (Figure 5e) illustrates the overlap of these upregulated GO terms, while the names of the remaining 12 co-enriched GO terms are listed in Figure 5f. This consistent enrichment pattern suggests that IFIT1 and RSAD2 may jointly regulate these biological functions in the context of GV and persistent HPV16 co-infection. In contrast, KEGG analysis revealed only one activated pathway, “Epithelial cell signaling in *Helicobacter pylori* infection,” and one repressed pathway, “Ribosome,” as shown in Supporting Information 2: Figure S2. Notably, *H. pylori* infection shares mechanistic similarities with HPV-related carcinogenesis, particularly in inflammation-driven and immune-regulatory processes. The enrichment of *H. pylori* infection-related signaling pathways (e.g., NF-*κ*B-mediated chronic inflammation, JAK-STAT immune evasion, and PI3K/AKT pro-proliferative pathways) likely reflects shared cancer-related pathways synergistically activated during GV and HPV16 co-infection [24–27]. Furthermore, the suppression of the ribosome pathway may be associated with HPV16 E6/E7-mediated regulation of ribosomal biogenesis and translation, as well as virus-induced host metabolic reprograming [28]. This effect could be exacerbated by the inflammatory environment triggered by GV, potentially disrupting host cell ribosomal function and protein synthesis.

### 3.5. Hub Genes Influencing Phenotypes Through Gene Interactions

Building on these findings, we further explored the potential impact of these hub genes on specific phenotypes. To this end, we organized the 17 significant GO terms and 2 KEGG pathways from the single-gene GSEA into eight phenotypic classifications (Supporting Information 1: Table S3). We then extracted the top 100 genes with the highest relevance scores for each phenotype from the GeneCards database as phenotypic genes (Supporting Information 1: Table S4). Correlation analyses were conducted between hub genes and these phenotypic genes to determine if hub genes influence these phenotypes through specific gene interactions. Notably, both IFIT1 and RSAD2 showed positive correlations with RIGI (*p* < 0.05). RIGI is linked to five phenotypes: viral assembly and packaging efficiency, viral replication rate, viral entry efficiency, regulated immune response, and interferon production. This finding suggests that in co-infection scenarios, IFIT1 and RSAD2 may influence these phenotypes through direct or indirect interactions with RIGI. Additionally, RSAD2 was positively correlated with CREB1 (*p* < 0.05), a gene associated with protein synthesis rate, regulated immune response, and inflammatory response. This suggests that RSAD2 may also affect these processes by interacting with CREB1 (Supporting Information 2: Figure S3).

### 3.6. Diagnostic Potential of Hub Genes in HPV16 and GV Infections

**Figure 6:**
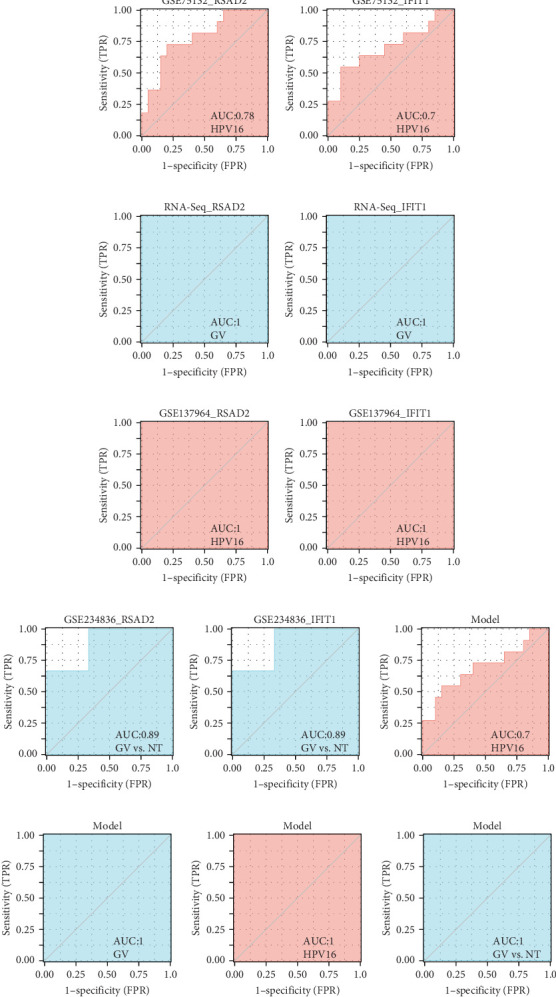
Diagnostic potential of hub genes in samples with HPV16 and GV infection. (a–d) ROC curve of the two shared genes in test (a, b) and validation datasets (c, d) for HPV16 and GV infection; (e–h) ROC curve of the multi-marker diagnostic model in test and validation datasets for HPV16 and GV infection; (b, f) GV infection in Siha; (d, h) GV infection in ECT1/E6E7. GV, *Gardnerella vaginalis*; ROC, receiver operating characteristic.

We validated the expression of the hub genes in additional HPV16 and GV infection datasets (GSE137964, GSE234836, and GSE129159), which also exhibited a significant downregulation (Table 1). To evaluate the diagnostic accuracy of two hub genes, RSAD2 and IFIT1, in predicting infection status, we performed ROC analysis on HPV16-infected and GV-infected samples across different datasets. The results showed that RSAD2 and IFIT1 effectively distinguished HPV16 infection from healthy controls and GV infection from untreated controls (AUC > 0.7), indicating significant diagnostic potential (Figure 6a–d). To improve the accuracy of predicting infection status, we used logistic regression to combine the expression levels of these two hub genes into a multi-biomarker diagnostic model. ROC analysis demonstrated that this model achieved an AUC of 0.70 in dataset GSE75132, 1.00 in the RNA-Seq dataset for GV infection, 1.00 in GSE137964, and 1.00 in GSE234836 (Figure 6e–h). These findings suggest that the multi-biomarker diagnostic model exhibits strong predictive performance for both HPV16 and GV infections.

### 3.7. Clinical Significance of Hub Genes in HPV-Related Cervical Lesion

**Figure 7:**
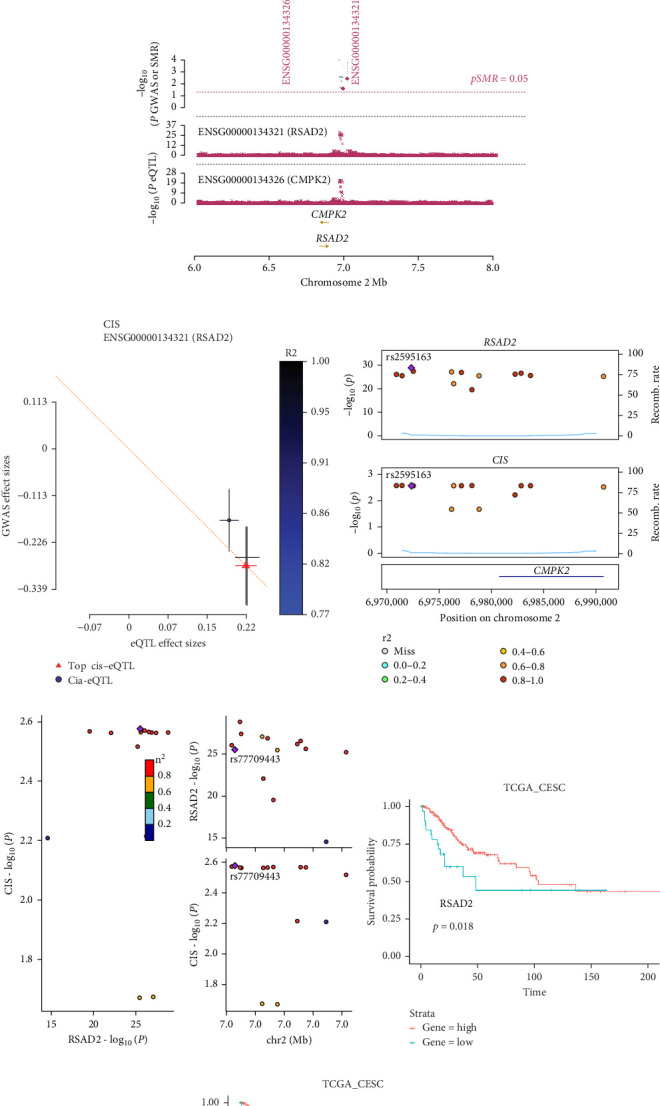
Analysis of the relationship between hub genes and cervical cancer onset and prognosis. (a) Scatter plot illustrates the association between the effects of SNPs on RSAD2 and their effects on CIS; (b) Leave-one-out sensitivity analysis for RSAD2 on CIS; (c) Pleiotropic associations between RSAD2 and CIS. Top plot: shows −log_10_ (*p* values) of SNPs from the CIS GWAS, with solid red rhombi indicating probes that passed the HEIDI test, middle plot: displays eQTL results, bottom plot: illustrates the locations of genes associated with the probes; (d) SMR indicating significant negative causal relationships between RSAD2 expressions and CIS onset (*p*_SMR_ < 0.05, *p*_HEIDI_ > 0.05). (e) Manhattan plot for the colocalization analysis of RSAD2 and CIS, where the diamond-shaped purple points represent the causal SNPs. (f) Comparison plot of loci for RSAD2 and CIS, with the diamond-shaped purple points indicating the lead SNP with the smallest *p* value. (g) Survival analysis of high-expression versus low-expression groups of RSAD2 in cervical cancer patients; (h) Survival analysis of high-expression versus low-expression groups of IFIT1 in cervical cancer patients. CIS, carcinoma in situ; RSAD2, radical S-adenosyl methionine domain containing 2; SNPs, single nucleotide polymorphisms.

Co-infection with GV and HPV16 often leads to a persistent HPV16 infection, which in turn drives the progression of cervical lesions. Therefore, this study explored the link between abnormal hub gene expression and different stages of cervical lesions, including LSIL, HSIL, CIS, and SCC. MR analysis (Supporting Information 1: Tables S6–9, Figure 7a,b, Supporting Information 2: Figure S4a,b) uncovered a causal connection between decreased RSAD2 expression and a higher risk of HSIL (IVW odds ratio [OR] = 0.80, 95% CI 0.68–0.94, *p*_adj_ = 7.96 × 10^−3^) and CIS (IVW OR = 0.71, 95% CI 0.50–0.99, *p*_adj_ = 4.93 × 10^−2^), with no evidence of heterogeneity (Cochran's Q *p* > 0.05) or pleiotropy (*p*_Global_ > 0.05, *p*_Intercept_ > 0.05). Reverse MR analysis (Supporting Information 1: Table S10) showed that cervical lesions do not influence RSAD2 expression (*p* > 0.05), ruling out reverse causation. Additional confirmation came from SMR and HEIDI tests (Supporting Information 1: Table S11, Figure 7c,d, Supporting Information 2: Figure S4c,d), which supported a correlation between RSAD2 expression levels and both HSIL (beta = −0.69, *p*_SMR_ = 1.02 × 10^−2^, *p*_HEIDI_ = 0.23) and CIS (beta = −1.27, *p*_SMR_ = 3.77 × 10^−3^, *p*_HEIDI_ = 0.19). Colocalization analysis further examined whether SNPs regulating RSAD2 expression impact cervical lesion development, revealing shared genetic effects between RSAD2 expression and CIS occurrence (Causal SNP = rs2595163, PPH4 = 0.62). This suggests that RSAD2 genetic variants affect both gene expression and CIS risk (Supporting Information 1: Table S12, Figure 7e,f). To evaluate the clinical relevance of these hub genes in HPV-related disease progression and prognosis, gene expression profiles and survival data from cervical cancer patients in the TCGA database were analyzed. Notably, lower RSAD2 expression appeared to be linked to worse cervical cancer prognosis (Figure 7g), while low IFIT1 expression was also associated with poorer survival, though its predictive power was not statistically significant (Figure 7h, *p* > 0.05). Together, these findings indicate that RSAD2 may play a critical role in cervical cancer triggered by GV and HPV16 co-infection.

### 3.8. Changes in Hub Gene Expression Patterns During the Progression From HPV Infection to Cervical Cancer

**Figure 8:**
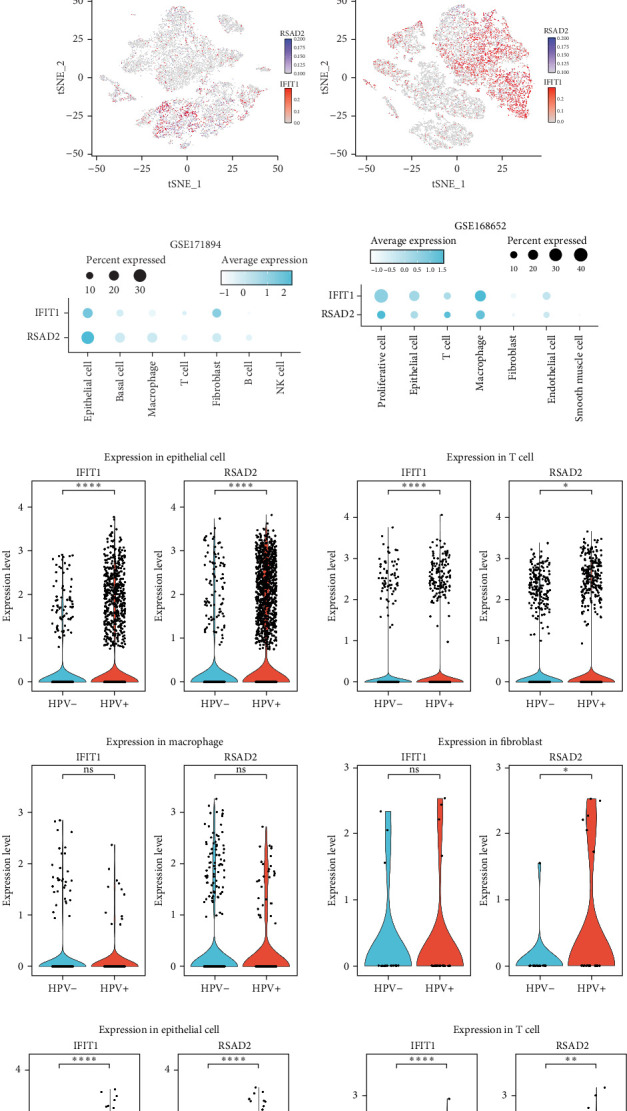
Single-cell transcriptomic characterization of cervical tissues from HPV16-infected patients and cervical cancer patients. (a) UMAP visualization of 14,253 cells from the HPV16 infection scRNA-seq dataset (GSE171894); (b) UMAP visualization of 22,234 cells from the cervical cancer scRNA-seq dataset (GSE168652); (c, d) UMAP visualization of cell clusters corresponding to different sample groups in HPV16-infected and cervical cancer patients; (e, f) UMAP visualization depicting the expression levels of two hub genes in HPV16-infected and cervical cancer patients; (g, h) Dot plots illustrating the expression of the two hub genes across various cell populations in HPV16-infected and cervical cancer patients; (i, j) Violin plots comparing the expression of the two hub genes in HPV16-positive vs. HPV-negative cases, as well as in cervical cancer versus normal tissues. UMAP, Uniform Manifold Approximation and Projection.

In the GSE171894 dataset (HPV16-positive vs. HPV16-negative groups), a total of 14,253 cells were identified and classified into seven distinct cell populations based on marker gene expression (Figure 8a). Similarly, in the GSE168652 dataset (cervical cancer vs. normal tissue groups), 22,234 cells were identified and annotated into the corresponding seven cell populations (Figure 8b). Uniform Manifold Approximation and Projection (UMAP) visualization revealed a slight upregulation trend in RSAD2 and IFIT1 expression in HPV16-positive samples compared to HPV16-negative samples, though the difference was not obvious (Figure 8c,e). However, in cervical cancer tissues IFIT1 expression was significantly upregulated, while RSAD2 showed no notable change (Figure 8d,f). Further analysis characterized the expression patterns of RSAD2 and IFIT1 across different cell types. In the HPV16-positive and HPV16-negative groups, RSAD2 was predominantly highly expressed in epithelial cells, whereas IFIT1 exhibited high expression in both epithelial cells and fibroblasts, with lower expression levels in immune cells (Figure 8g). In the cervical cancer vs. normal tissue groups, RSAD2 and IFIT1 expression was significantly upregulated in macrophages and T cells, with additional expression observed in epithelial cells and proliferative cells (Figure 8h). More specifically, in the HPV16 infection group, RSAD2 was significantly upregulated in epithelial cells, T cells, and fibroblasts, but showed no notable changes in macrophages (Figure 8i). IFIT1 was significantly upregulated in epithelial cells and T cells, with no obvious changes in macrophages or fibroblasts (Figure 8i). In contrast, in cervical cancer tissues, both RSAD2 and IFIT1 were markedly elevated in macrophages, and RSAD2 was significantly downregulated in fibroblasts (Figure 8j). These findings suggest that during the transition from persistent HPV16 infection to cervical cancer, the upregulation of RSAD2 and IFIT1 in macrophages may indicate a shift in immune status, potentially linked to immune evasion and chronic inflammation. This altered expression pattern may play a critical role in the progression of cervical lesions, particularly in the context of co-infection.

### 3.9. Confirming Hub Gene Expression In Vitro

In vitro validation demonstrated that IFIT1 and RSAD2 expression levels were significantly reduced in HPV16-positive Siha and Caski cells compared to HPV-negative HaCaT cells (Supporting Information 2: Figure S5a,c), confirming that persistent HPV16 infection downregulates these genes. Additionally, a similar downregulation was observed in Siha, Caski, and HaCaT cells co-cultured with GV, indicating that GV infection also downregulates these genes (Supporting Information 2: Figure S5b,d). These findings are consistent with sequencing results.

## 4. Discussion

This study integrated transcriptomic data from GV and persistent HPV16 infections to explore the shared pathogenic mechanisms and potential biomarkers during co-infection. The analysis revealed that the interferon-induced genes IFIT1 and RSAD2 play central roles in the pathological process of co-infection. Further investigations were conducted on their upstream transcriptional regulatory networks (including transcription factors and ceRNA networks), downstream changes in related biological functions and phenotypes, carcinogenic potential, and clinical diagnostic and predictive value, comprehensively elucidating the molecular basis in the co-infection context. These findings not only unveil the complex molecular interactions between GV and HPV16 co-infection but also provide valuable biomarkers for further research.

Building on the molecular insights, it is essential to discuss the experimental models used to study these mechanisms. The monolayer cell model provides a direct and effective tool for studying the impact of GV infection on cervical epithelial cells, and its utility has been widely validated by previous research. For instance, co-culture experiments with GV and cells have demonstrated that GV can efficiently adhere to and invade cervical epithelial cells (e.g., HeLa, Siha) and vaginal epithelial cells (e.g., VK2/E6E7), a process mediated by protein adhesins and facilitated by phagocytosis (internalization) for colonization within host cells [29, 30]. Furthermore, studies using the monolayer cell model have revealed that GV induces apoptosis and necrosis through the secretion of vaginolysin toxin and activates the TLR2/NF-*κ*B signaling pathway, leading to the overexpression of pro-inflammatory cytokines such as IL-6, IL-8, and TNF-*α*, which ultimately compromises the cervical and vaginal epithelial barrier functions [31, 32]. These findings collectively affirm the feasibility and significance of the monolayer cell model in elucidating the mechanisms of GV infection.

Nevertheless, our initial attempts to apply a direct co-culture model in the context of GV-HPV co-infection revealed significant limitations. Preliminary experiments indicated that direct contact resulted in rapid cell death, hindering the accurate capture of the dynamic pathogenic processes underlying GV-HPV co-infection. To address this limitation, we refined the model by introducing an invasion chamber as a physical barrier, enabling indirect interactions between GV and cells via soluble factors in the culture medium (e.g., metabolites, extracellular vesicles, and secreted proteins). In the vaginal environment of healthy women, epithelial cells are typically covered by a layer of mucus, which effectively serves as a physical barrier between bacteria and the epithelial cells. Consequently, although there is close interaction between bacteria and epithelial cells, this interaction is predominantly mediated indirectly through bacterial secretions such as metabolites, extracellular vesicles, and other soluble factors, rather than direct cell-to-cell contact. Moreover, in the context of HPV-infected epithelial cells, their infection processes and pathological changes may similarly be regulated by these soluble factors within the microenvironment. Thus, the use of an indirect co-culture model is not only theoretically feasible but also more reflective of the in vivo conditions.

Our study also highlighted the pivotal role of hub gene RSAD2 and IFIT1 in the context of co-infection. The IFIT1 gene, located at 10q23, is induced by type 1 interferons or viral infections [33] and exhibits extensive antiviral activities as well as anti-inflammatory effects. The anti-inflammatory mechanisms of IFIT1 are not yet fully elucidated [34]. Research indicates that IFIT1 interacts with the Sin3A-HDAC complex to inhibit transcription factor recruitment at inflammatory cytokine sites, effectively reducing the expression of pro-inflammatory cytokines like TNF-*α* [35]. Furthermore, overexpression of IFIT1 enhances cell survival by suppressing JNK pathway activation, thus preventing cell apoptosis [36]. Additionally, IFIT1 helps to alleviate inflammation-related damage by downregulating inflammatory factors via the p38 MAPK signaling pathway [37]. Additionally, research by Fan et al. [38] has shown that the rs303212 single nucleotide polymorphism variation at the IFIT1 gene locus on chromosome 10 is suggestively associated with the relative abundance of common vaginal bacteria, including *Lactobacillus*, *Gardnerella*, and *Actinomycetes*. This highlights the potential impact of IFIT1 on the vaginal microbiome, including in conditions involving GV. These findings support the results of our study, which demonstrate a significant downregulation in IFIT1 expression within the GV co-culture system.

In antiviral responses, IFIT1 can recognize viral RNA with 2′-O unmethylated RNA and 5′-PPP, inhibiting viral protein translation by binding to initiation factors such as eIF3 at specific sites [39]. This study observed a significant downregulation of IFIT1 expression in populations with persistent HPV16 infections and HPV16-positive cells, suggesting a potential state of immune suppression. Correspondingly, Reiser et al. [40] reported suppressed expression of the antiviral gene IFIT1 in HPV16-positive keratinocytes. However, contrasting findings were reported by Klymenko et al. [41] in HPV16-positive NIK cells, where HPV16 infection was actually found to upregulate IFIT1 expression. This discrepancy may stem from differences in the experimental and control cell lines used in the studies. Moreover, the timing of the assays post-viral transfection was not explicit. Initially, IFIT1 production might surge temporarily to combat infection, but as viral plasmids stabilize within the cells over time, leading to the establishment of a mature immune evasion mechanism, IFIT1 expression could be suppressed. Future research needs to further investigate the precise mechanisms behind this phenomenon.

RSAD2, located on human chromosome 2p25.2 [42], is also an interferon-stimulated gene (ISG) with anti-inflammatory and antiviral functions. The role of RSAD2 in inflammatory responses is not fully understood, yet it is known that knockdown of RSAD2 significantly reduces TNF-*α* production under LPS stimulation, indicating its crucial regulatory role in inflammation [43, 44]. In terms of antiviral activity, RSAD2 functions through the enzymatic activity of its radical SAM (S-adenosylmethionine) domain. This enzyme is capable of releasing a highly reactive 5′-deoxyadenosyl radical from the SAM molecule, which can disrupt molecular components essential for viral replication [45]. RSAD2 also inhibits viral replication by altering intracellular metabolic pathways, such as affecting the levels of intracellular triphosphonucleotides [46]. This study is the first to find that RSAD2 expression is suppressed in both GV infection and persistent HPV16 infection, suggesting a significant role of RSAD2 in co-infection scenarios. However, further research is required to substantiate these conclusions given the absence of similar literature on this analysis.

To uncover the direct regulatory mechanisms of IFIT1 and RSAD2, this study employed the RcisTarget R package for transcription factor enrichment analysis, identifying potential binding nucleotide sequences of transcription factors targeting these genes. Beyond transcription factor regulation, gene expression is also influenced by noncoding RNAs. Therefore, a ceRNA network involving these core genes was constructed. miRNA enrichment analysis on IFIT1 and RSAD2 revealed 122 miRNAs capable of co-regulating both genes. A search in the HMDD database revealed that hsa-mir-34a [47] and hsa-mir-654 [48], exhibit strong associations with HPV infection. Their mature forms, hsa-miR-34a-3p and hsa-miR-654-5p, can directly regulate the expression of IFIT1 and RSAD2, suggesting that they may be non-coding RNA molecules potentially influenced by co-infection and are worth further study.

To further understand the potential pathogenic mechanisms in co-infection processes, this study employed single-gene GSEA to investigate the biological pathways regulated by hub genes. The analysis revealed enrichment in antiviral response, immune regulation, viral replication, viral lifecycle, and type I interferon production in both GV infection and HPV16 persistent infection groups, indicating that type I interferon-induced antiviral activity plays a crucial role in the co-infection process. Additionally, activation of transcription initiation and protein binding modulation was observed, which may be associated with the regulation of the HPV viral lifecycle and the modulation of viral gene expression within host cells [49]. Moreover, signaling pathways related to *H. pylori* infection in epithelial cells, including activation of the NF-*κ*B pathway and regulation of the PI3K-Akt pathway and JAK-STAT-driven immune escape, were also enriched [24–27]. This suggests that these pathways may act as common reactive routes triggered during co-infection. The suppression of the ribosomal pathway, which plays a significant role in bacterial physiology [50], might be associated with the anti-inflammatory functions of the hub genes.

Single-gene GSEA analysis indicates that IFIT1 and RSAD2 regulate multiple biological processes during co-infection. Correlation analysis further suggests that these hub genes may interact with RIGI to impact viral assembly and packaging efficiency, viral replication rate, viral entry efficiency, regulated immune response, and interferon production. RIGI, a pattern recognition receptor, can recognize HPV16 RNA and activate antiviral immune responses [51, 52]. However, HPV16 proteins E6 and E7 might suppress RIGI function to evade host immune surveillance [53]. Additionally, RIGI plays a significant role in bacterial infection recognition, aiding in the defense against a broader spectrum of pathogens [51]. Furthermore, RSAD2, either directly or indirectly, may cooperate with CREB1 to regulate protein synthesis rate, immune response, and inflammatory response. Research indicates that CREB1 plays a role in regulating the expression of Fc gammaRIIA, a receptor expressed on neutrophils and monocytes, which is crucial for combating bacterial infections [54]. Similarly, RSAD2, either directly or indirectly, may also collaborate with CDC42 to modulate immune response, cell migration, and proliferation.

ROC analysis and survival analysis indicate that RSAD2 and IFIT1 hold potential value in the diagnosis and prognostic evaluation of HPV-related lesions. Previous studies have reported a surface-enhanced Raman scattering (SERS)-based nanoprobes technology for detecting RSAD2 RNA in the diagnosis of respiratory viral infections [55]. As an ISG, IFIT1 has been implicated in the progression of HPV-related diseases, including HPV-associated head and neck squamous cell carcinoma (HNSCC), cervical cancer, and other HPV-related malignancies [40, 56–58]. However, in contrast to RSAD2, no standardized detection methods for IFIT1-based viral diagnostics currently exist. Therefore, future research should explore novel molecular detection technologies based on IFIT1, such as SERS, CRISPR-dx, or nanosensors, to enable early screening and precise prognostic assessment of HPV-related diseases.

Although no studies have directly investigated RSAD2 in the treatment of HPV-related diseases, existing research suggests that RSAD2 influences viral replication and infection persistence in other viral infections, such as HIV and measles virus [59, 60], providing insights into its potential therapeutic applications in HPV-related diseases. Additionally, studies have demonstrated that IFIT1 can inhibit the HPV E1 protein, making it a potential target for antiviral drug development [40]. Interferon therapy may enhance HPV clearance by upregulating IFIT1 expression [57], while methylation inhibitors can restore IFIT1 function, thereby boosting the host's antiviral immunity. Notably, HPV may evade immune detection by suppressing IFIT1 expression [58]. Therefore, restoring IFIT1 expression could represent a novel strategy to counteract viral immune evasion and enhance anticancer treatment efficacy.

Through MR, SMR, and colocalization analyses, we validated the causal relationship between the expression of the hub gene RSAD2 and CIS. RSAD2 has already been identified as a significant factor affecting the progression and prognosis of various cancers [61–63] including colorectal and breast cancer. In colorectal cancer, RSAD2 is a key component of the tumor immune microenvironment and serves as a prognostic predictor [62]. In breast cancer, RSAD2, along with HERC5 and CCL8, was identified as a crucial gene impacting prognosis through gene co-expression network analysis [63]. Our research further confirms the critical role of RSAD2 in the progression of cervical cancer, suggesting it as a potential new target for cervical cancer prevention and treatment.

The survival analysis from the TCGA database further emphasizes the clinical relevance of this study, showing that lower RSAD2 expression is associated with worse cervical cancer prognosis. This finding is consistent with previous studies that indicate RSAD2 functions as a tumor suppressor by enhancing immune responses and inhibiting oncogenic pathways [64]. Our findings support the hypothesis that RSAD2 could serve as a prognostic biomarker and a potential therapeutic target for cervical cancer.

Moreover, single-cell transcriptomic data analysis in this study revealed the dynamic expression patterns of RSAD2 and IFIT1 across different cell types, particularly during HPV infection and cervical cancer progression. Notably, RSAD2 expression was significantly increased in macrophages and T cells in cervical cancer tissues, which may reflect changes in the tumor immune microenvironment [59, 65]. Previous research has suggested that RSAD2 influences tumor immune status via interferon-stimulated pathways [66]. The observed upregulation of RSAD2 in immune cells, particularly macrophages, may represent an adaptive immune response to persistent HPV infection and chronic inflammation—both of which are hallmarks of cervical cancer progression.

Further analysis of IFIT1 expression suggests its complex role in immune regulation during cervical cancer progression. Although HPV infection upregulates IFIT1 expression in epithelial cells and T cells, its expression is markedly elevated in macrophages within cervical cancer tissues. As a key ISG, IFIT1 plays a crucial role in antiviral immune responses and tumor progression [67]. The increased expression of IFIT1 in macrophages may reflect an attempt to counteract immune suppression within the tumor microenvironment. However, this study did not find IFIT1 to be a significant prognostic predictor for cervical cancer, suggesting that its role may be highly context-dependent and warranting further investigation [68].

This study elucidates the molecular basis of epithelial cell functional changes regulated by GV and HPV16 infection, providing robust theoretical support for further exploration of how microbial dysbiosis combined with persistent HPV infection accelerates cervical lesion progression. Nevertheless, certain limitations remain. Firstly, the GV-cell co-culture model employed in this study can only simulate the effects of GV overgrowth on cervical epithelial cells and does not fully capture the complex interactions of diverse microbial flora in the real vaginal environment. However, this co-culture model enables us to deeply investigate changes in the core mechanisms of epithelial cells under specific microbial conditions. Future validation of our findings is warranted in populations with high GV abundance and persistent HPV16 infection. Secondly, the HPV16 infection GEO dataset used lacks relevant clinical information, leaving it uncertain whether the infection ultimately leads to carcinogenesis. Thus, caution is required when interpreting the results. Future studies should employ additional molecular biology experiments—such as regulating key gene expression and examining changes in HPV16 replication, immune cell function, cytokine activity, and epithelial cell carcinogenic phenotypes—to further substantiate our findings.

## 5. Conclusions

Our study identifies IFIT1 and RSAD2 as key regulators in GV and persistent HPV16 co-infection. Differential expression and enrichment analyses revealed 74 co-regulated genes primarily involved in immune defense, pathogen response, and metabolic pathways. Network analyses, including transcription factor and ceRNA evaluations, further confirmed that IFIT1 and RSAD2 are central to interferon-mediated antiviral responses. GSEA reinforced their roles in modulating host-pathogen interactions. Clinically, MR and colocalization analyses demonstrated that reduced RSAD2 expression is causally linked to an increased risk of cervical lesions. Single-cell transcriptomics and in vitro experiments validated the dynamic expression changes of these hub genes during infection progression. Additionally, ROC and logistic regression analyses revealed that the combined expression of IFIT1 and RSAD2 effectively distinguishes HPV16 and GV infections, underscoring their diagnostic value. Collectively, these findings establish a robust molecular framework for understanding GV and HPV16 co-infection pathogenesis and highlight IFIT1 and RSAD2 as promising diagnostic biomarkers and therapeutic targets in HPV-related diseases.

## Figures and Tables

**Table 1:** Detailed information of the five included datasets.

Datasets	Gene	Group	Experimental sample size	Control sample size	Type of samples	*R* package	Log2FC	*p*-Value	Padj
GSE75132^a^	RSAD2	Persistent HPV16 infection vs. HPV negative	20	11	Cervix	limma	−0.73	0.04	0.29
	IFIT1	Persistent HPV16 infection vs. HPV negative	20	11	Cervix	limma	−1.11	0.02	0.22

RNA-Seq^a^	RSAD2	GV live bacteria vs. live bacteria non-treated control	6	6	Siha	DESeq2	−1.54	<0.001	<0.001
	IFIT1	GV live bacteria vs. live bacteria non-treated control	6	6	Siha	DESeq2	−1.94	<0.001	<0.001

GSE129159^b^	RSAD2	Productive HPV16 infection vs. HPV negative	15	15	Cervix foreskin and tonsil organotypic raft	limma	−0.26	0.03	0.37
	IFIT1	Productive HPV16 infection vs. HPV negative	15	15	Cervix foreskin and tonsil organotypic raft	limma	−0.71	0.22	0.70

GSE234836^b^	RSAD2	GV live bacteria vs. live bacteria non-treated control	3	3	Ect1/E6E7	DESeq2	−1.05	<0.001	<0.01
	IFIT1	GV live bacteria vs. live bacteria non-treated control	3	3	Ect1/E6E7	DESeq2	−1.45	<0.001	<0.01
	RSAD2	GV live bacteria vs. live bacteria non-treated control	3	3	VK2/E6E7	DESeq2	−1.16	0.11	0.29
	IFIT1	GV live bacteria vs. live bacteria non-treated control	3	3	VK2/E6E7	DESeq2	−1.66	<0.001	<0.001

GSE160008^b^	RSAD2	HPV16 positive vs. HPV negative	3	1	Head and neck squamous cell carcinoma	DESeq2	−4.03	<0.001	0.09
	IFIT1	HPV16 positive vs. HPV negative	3	1	Head and neck squamous cell carcinoma	DESeq2	−4.47	<0.001	0.01

*Note:* Padj, The *p*-value adjusted using the false discovery rate (FDR) correction. Log2FC, Log2-fold change. Positive values indicate upregulation; negative values indicate downregulation.

Abbreviation: GV, *Gardnerella vaginalis*.

^a^This dataset is used to identify differentially expressed genes.

^b^This dataset is used to validate hub genes.

## Data Availability

Part of the data utilized in this study is accessible on public data platforms (see Supporting Information 1: Table S5 and Table 1 for details). The RNA sequencing data regarding GV infection reported in this paper are openly available in the Sequence Read Archive (SRA) at https://dataview.ncbi.nlm.nih.gov/object/PRJNA1217577, reference number PRJNA1217577.

## Nomenclature

*G. vaginalis*: *Gardnerella vaginalis*
*PPI*: Protein–protein interaction
*GSEA*: Gene set Enrichment Analysis
*HR-HPV*: High-risk human papillomaviruses
*CIN*: Cervical intraepithelial neoplasia
*DEGs*: Differentially expressed genes
*GO*: Gene ontology
*KEGG*: Kyoto Encyclopedia of Genes and Genomes
*NES*: Normalized Enrichment Score
*ceRNA*: Competing endogenous RNA
*AUC*: Area under the curve
*MR*: Mendelian Randomization
*IVW*: Inverse variance weighting
*SMR*: Summary-data-based mendelian randomization
*RT-PCR*: Real-time PCR
*LSIL*: Low-grade squamous intraepithelial lesion
*HSIL*: High-grade squamous intraepithelial lesion
*CIS*: Carcinoma in situ
*SCC*: Squamous cell carcinoma.

## Supporting Information

Supporting Information 1 Table S1：Top 10 common genes identified by 9 CytoHubba algorithms. Table S2：Transcriptional enrichment analysis results of hub genes. Table S3：Biological phenotypes involved in single-gene GSEA intersection items of hub genes. Table S4：Top 100 phenotype-associated genes from GeneCards. Table S5：Characteristics of GWAS datasets used in the study. Table S6：The instruments of exposure for Mendelian Randomization. Table S7：Mendelian Randomization analysis: RSAD2 impact on cervical lesions of different grades. Table S8：Mendelian Randomization analysis: IFIT1 impact on cervical lesions of different grades. Table S9：Analysis of genetic variants using heterogeneity tests, pleiotropy tests, and MR-PRESSO outcomes. Table S10：Reverse Mendelian Randomization analysis: the influence of cervical lesion severity on RSAD2 expression. Table S11：Summary-based Mendelian randomization (SMR) analysis from RSAD2 expression to cervical lesions of different grades (*p*_SMR_<0.05& *p*_HEIDI_＞0.05). Table S12：Colocalization analysis between gene expression and cervical lesions of different grades (PPH4 > 0.5). Table S13：Primer sequences of hub genes.

Supporting Information 2 Figure S1: miRNA regulatory network of hub genes. Figure S2: Single-gene GSEA enrichment analysis of hub genes (KEGG gene sets). Figure S3: Correlation analysis between hub genes and phenotypic gene sets. Figure S4: MR, SMR analysis of the causal relationship between RSAD2 and HSIL. Figure S5: Experimental validation of hub gene expression in vitro.
