# Supplementary material for: Integrative Analysis of Shared Pathogenic Genes and Potential Mechanisms in Gardnerella vaginalis and Persistent HPV16 Infection
Source: Mediators Inflamm. 2025 Jun 5;2025:2582989. doi: 10.1155/mi/2582989 (PMC12162162; doi:10.1155/mi/2582989)
Supplement: Supporting Information 2 — Figure S1: miRNA regulatory network of hub genes. Figure S2: Single-gene GSEA enrichment analysis of hub genes (KEGG gene sets). Figure S3: Correlation analysis between hub genes and phenotypic gene sets. Figure S4: MR, SMR analysis of the causal relationship between RSAD2 and HSIL. Figure S5: Experimental validation of hub gene expression in vitro. [file 2582989.f2.docx]

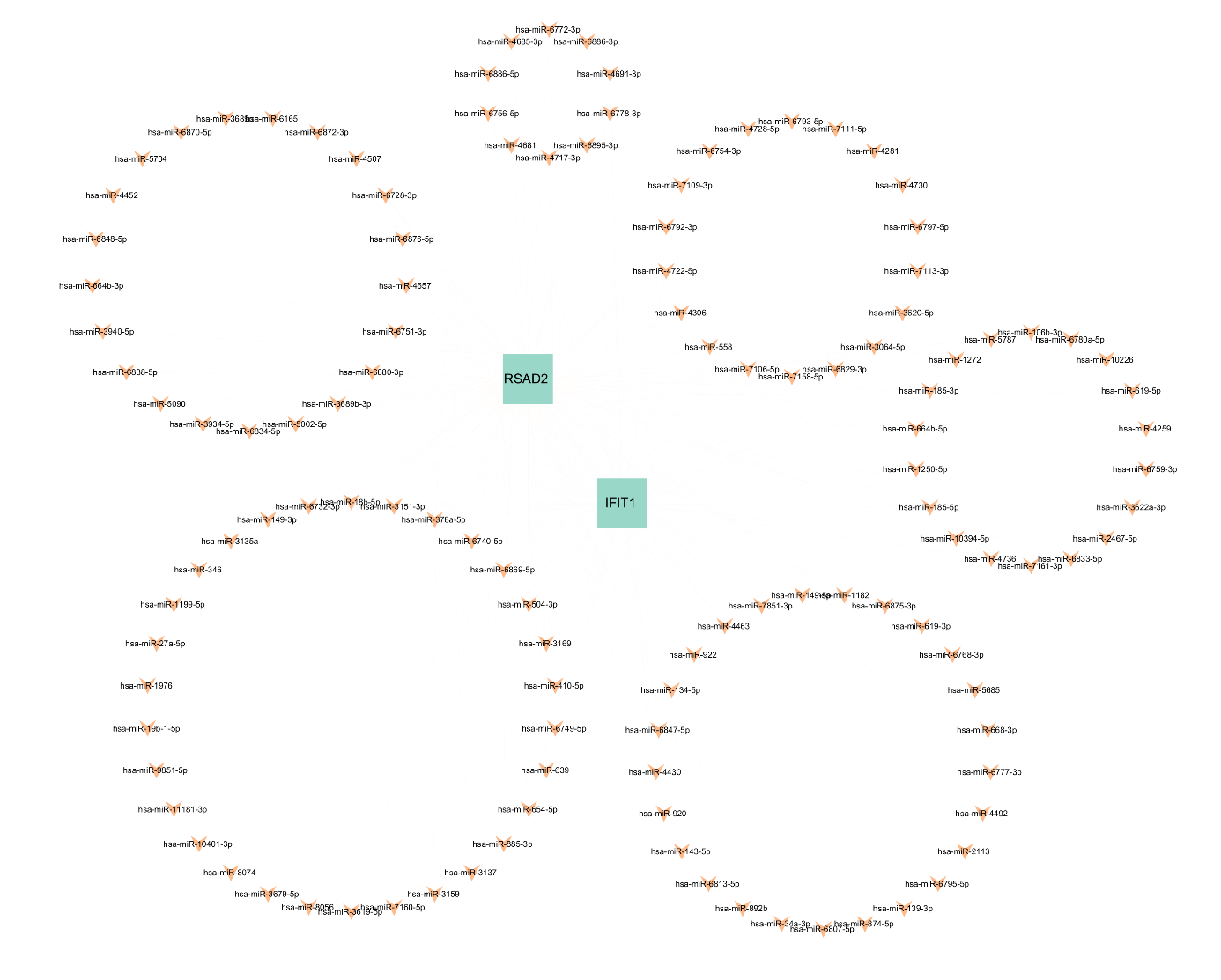
**Fig S1: miRNA regulatory network of hub genes**

miRNA network of hub genes, where yellow represents miRNAs and green represents mRNAs.


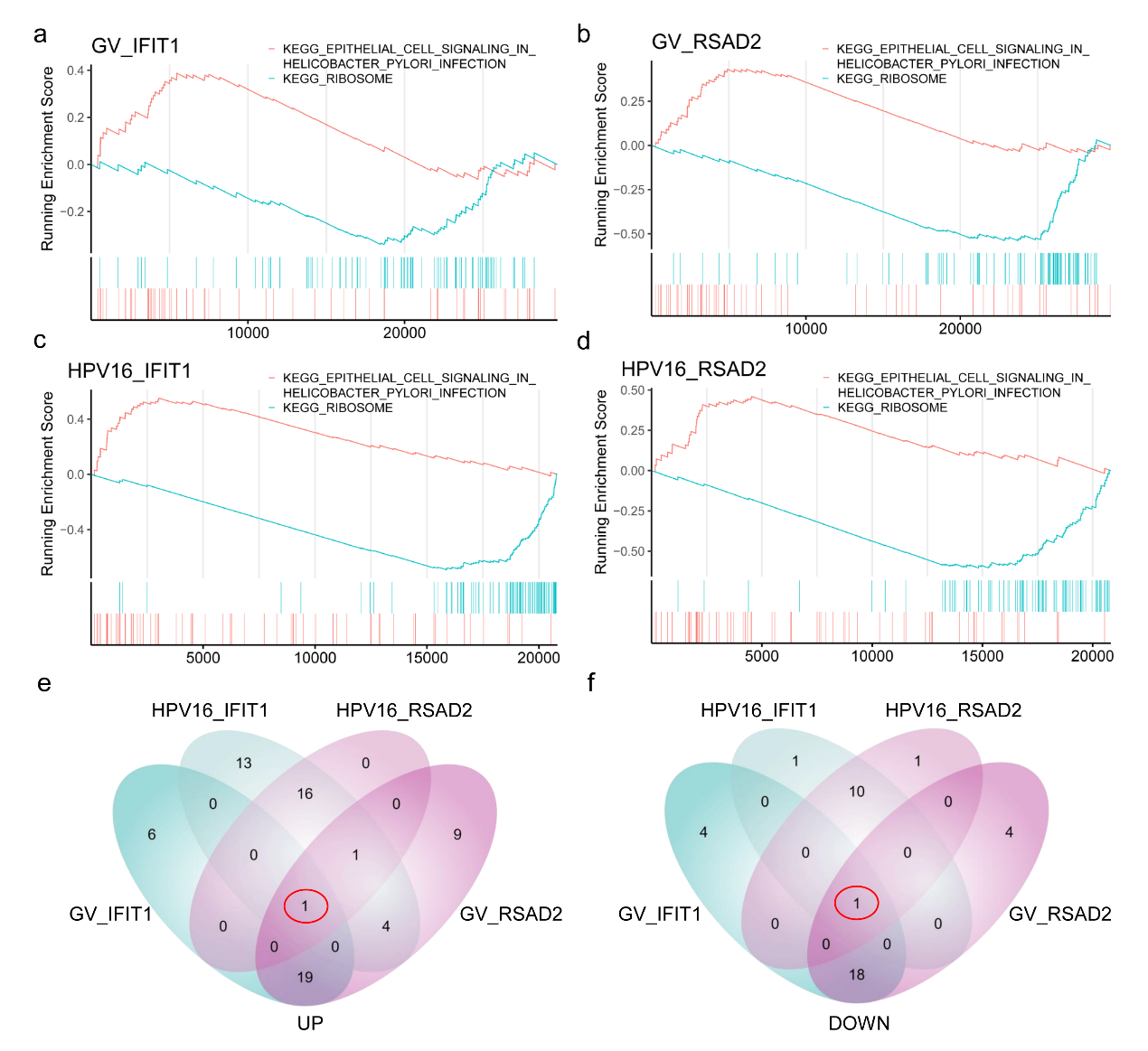


**Fig S2: Single-gene GSEA enrichment analysis of hub genes (KEGG gene sets)**

GSEA enrichment analysis of IFIT1 in the GV infection dataset (a) and the persistent HPV16 infection dataset (b), with two KEGG terms co-enriched by IFIT1 and RSAD2 across both datasets highlighted in the plots; GSEA enrichment analysis of RSAD2 in the GV infection dataset (c) and the persistent HPV16 infection dataset (d), with the same two KEGG terms highlighted; e. Venn diagram of KEGG terms commonly enriched by both IFIT1 and RSAD2 across the two datasets.


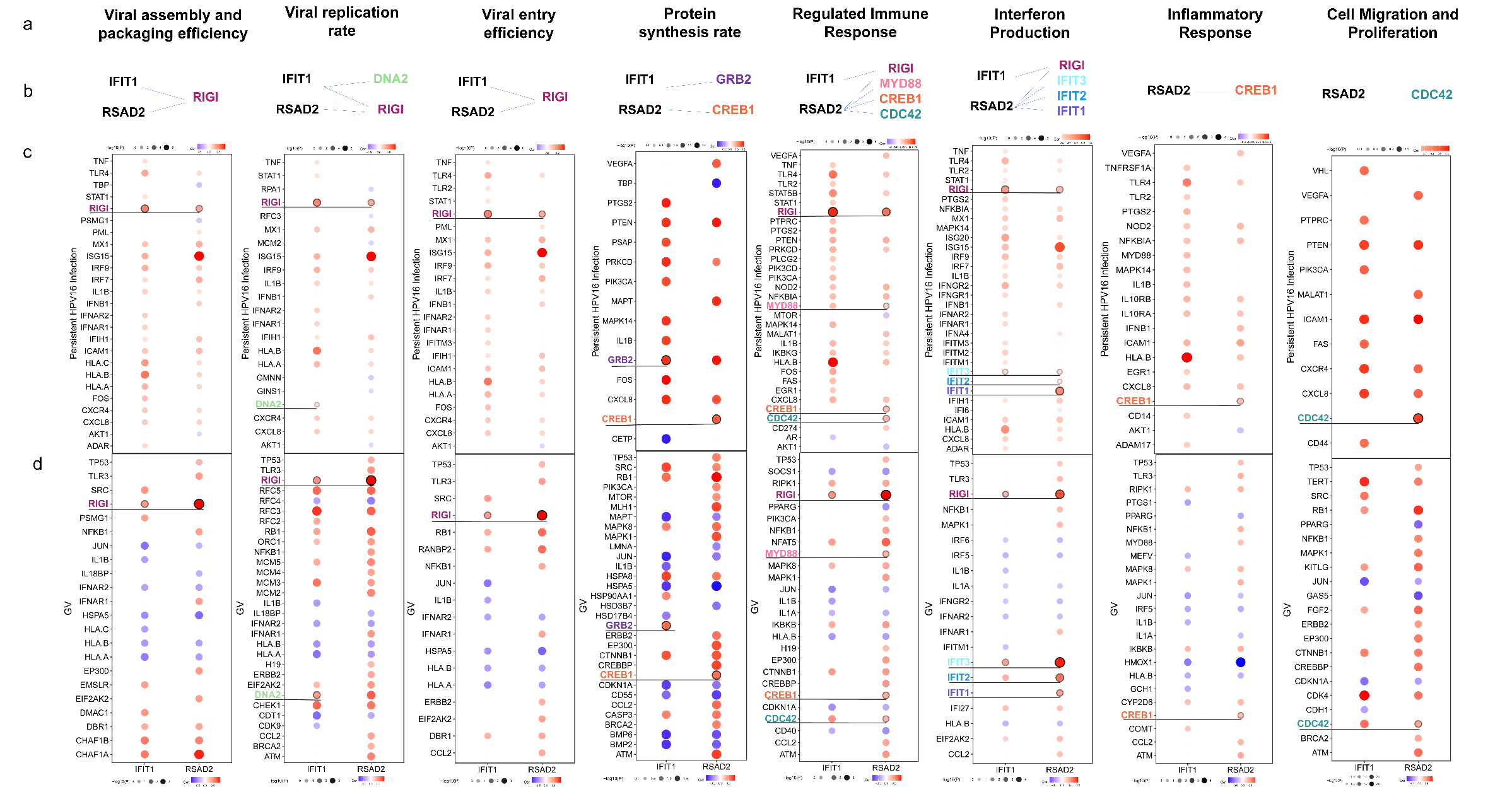


**Fig S3: Correlation analysis between hub genes and phenotypic gene sets**

Phenotypic gene set composed of the top 100 genes related to phenotype by Relevance score from the GeneCards database. **a.** Names of eight different phenotypes; **b.** Pairs of hub genes and phenotypic gene set genes with significant correlations; **c.** Bubble chart showing the correlation between hub genes and the phenotypic gene set in the persistent HPV16 infection dataset; **d.** Bubble chart showing the correlation between hub genes and the phenotypic gene set in the GV infection dataset.


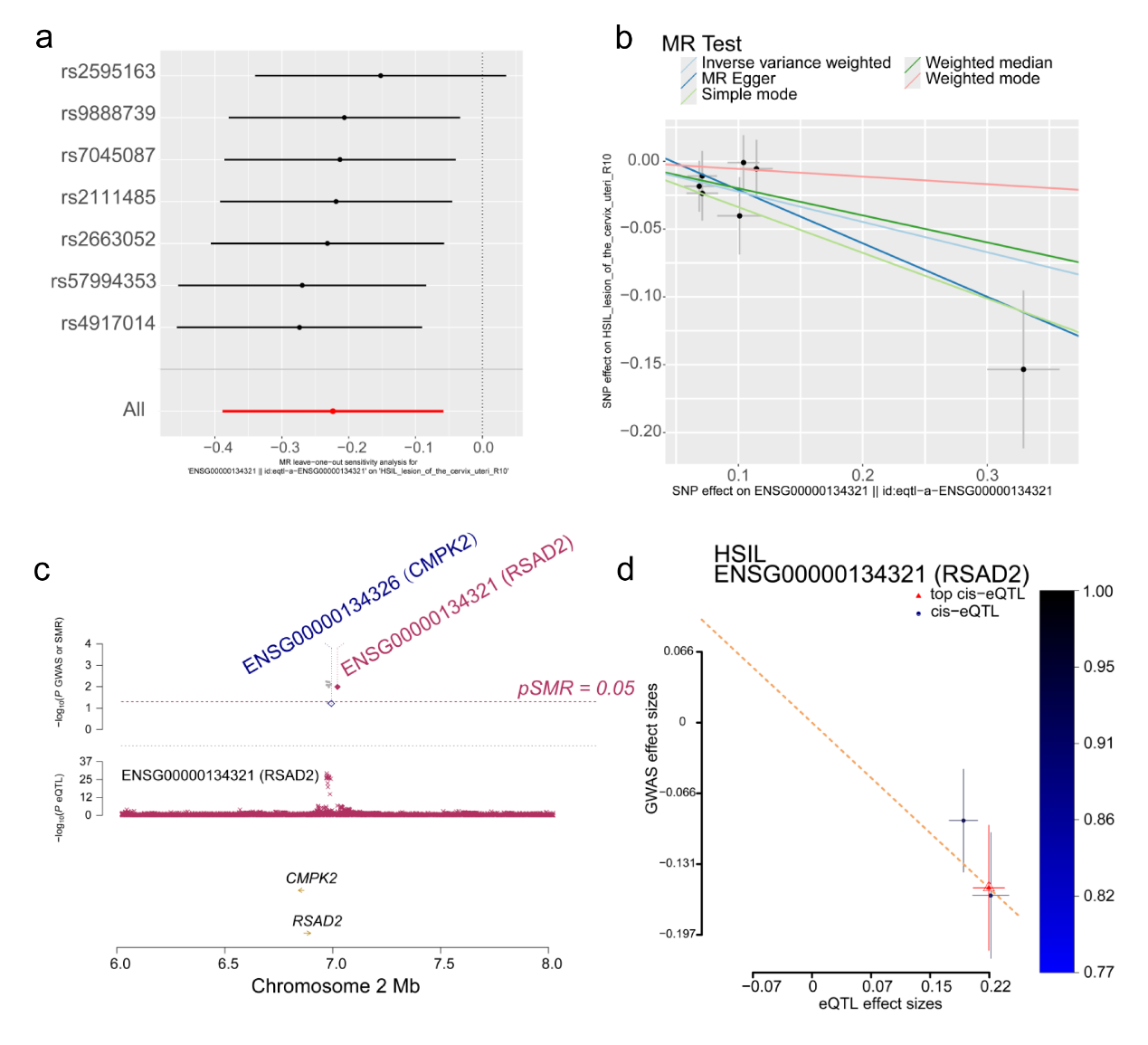


**Fig S4 MR, SMR analysis of the causal relationship between RSAD2 and HSIL**

**a.** Scatter plot illustrates the association between the effects of SNPs on RSAD2 and their effects on HSIL; **b.** Leave-one-out sensitivity analysis for RSAD2 on HSIL; c. Pleiotropic associations between RSAD2 and HSIL. Top plot: Gray dots show the −log10(*P* values) of SNPs from the GWAS for HSIL; Rhombuses represent the −log10(*P* values) from SMR testing, with solid rhombuses indicating probes that pass the HEIDI test and hollow rhombuses marking those that do not, middle plot: displays eQTL results, bottom plot: illustrates the locations of genes associated with the probes; **d**. SMR indicating significant negative causal relationships between RSAD2 expressions and HSIL onset (*P*_SMR_ < 0.05, *P*_HEIDI_ > 0.05). HSIL: high-grade squamous intraepithelial lesion**.**


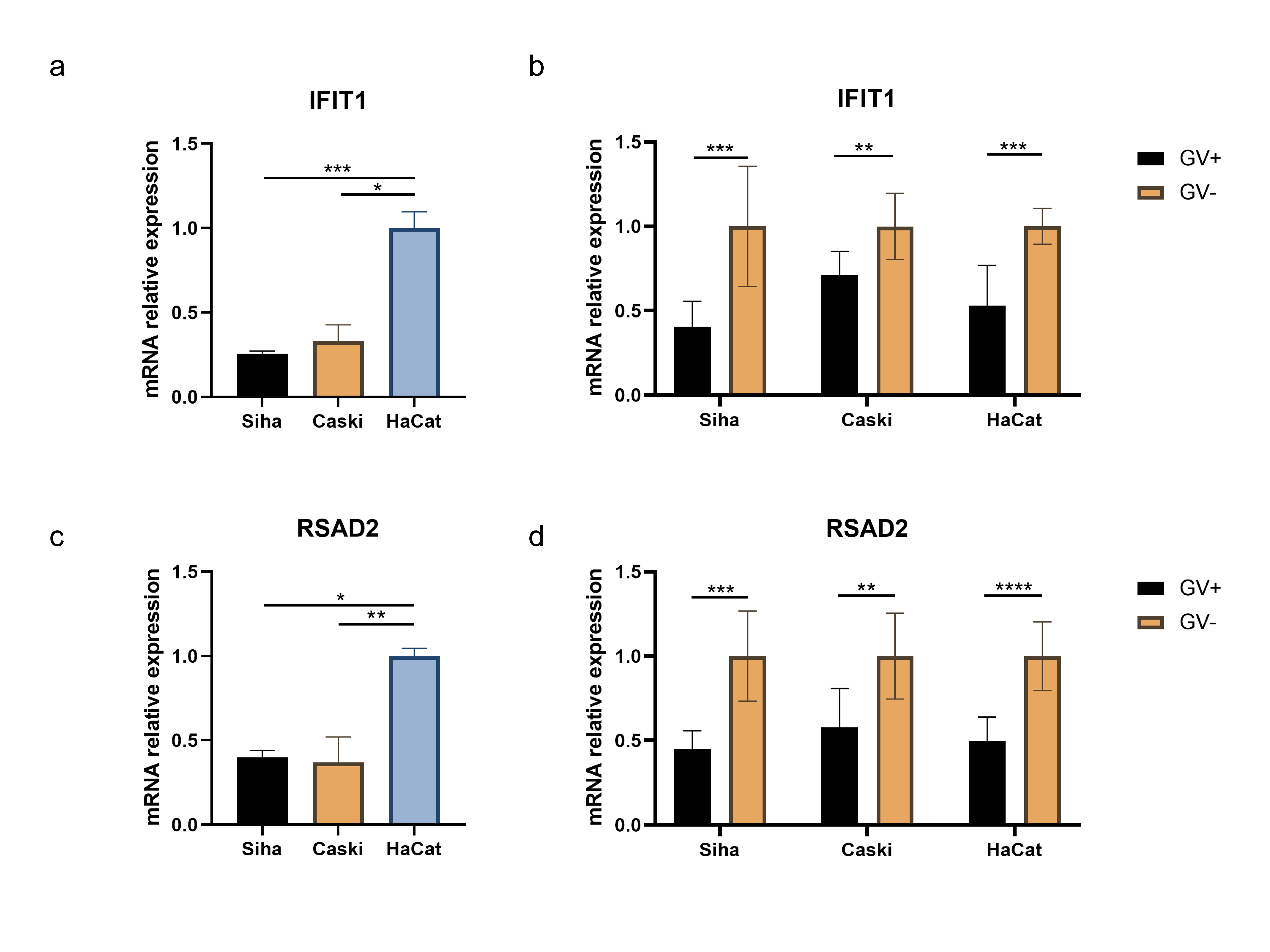


**Fig S5 Experimental validation of hub gene expression in vitro**

a. Relative expression of IFIT1 mRNA in HPV16 positive Siha and Caski cells compared to HPV negative HaCaT cells. b. Relative expression of IFIT1 mRNA in Siha, Caski, and HaCaT cells following GV infection versus uninfected controls. c. Relative expression of RSAD2 mRNA in HPV16 positive Siha and Caski cells compared to HPV negative HaCaT cells. d. Relative expression of RSAD2 mRNA in Siha, Caski, and HaCaT cells following GV infection versus uninfected controls. Each trial was performed 3 times for statistical comparison. *P < 0.05, **P < 0.01, ***P < 0.001, ****P < 0.0001.
